# Herpesviruses Serology Distinguishes Different Subgroups of Patients From the United Kingdom Myalgic Encephalomyelitis/Chronic Fatigue Syndrome Biobank

**DOI:** 10.3389/fmed.2021.686736

**Published:** 2021-07-05

**Authors:** Tiago Dias Domingues, Anna D. Grabowska, Ji-Sook Lee, Jose Ameijeiras-Alonso, Francisco Westermeier, Carmen Scheibenbogen, Jacqueline M. Cliff, Luis Nacul, Eliana M. Lacerda, Helena Mouriño, Nuno Sepúlveda

**Affiliations:** ^1^Departamento de Estatística e Investigação Operacional, Faculdade de Ciências, Universidade de Lisboa, Lisboa, Portugal; ^2^CEAUL–Centro de Estatística e Aplicações da Universidade de Lisboa, Lisboa, Portugal; ^3^Department of Biophysics, Physiology, and Pathophysiology, Medical University of Warsaw, Warsaw, Poland; ^4^Department of Infection Biology, Faculty of Infectious and Tropical Diseases, London School of Hygiene and Tropical Medicine, London, United Kingdom; ^5^Department of Statistics, Mathematical Analysis and Optimization, Universidade de Santiago de Compostela, Santiago de Compostela, Spain; ^6^Institute of Biomedical Science, Department of Health Studies, FH Joanneum University of Applied Sciences, Graz, Austria; ^7^Centro Integrativo de Biología y Química Aplicada (CIBQA), Universidad Bernardo O'Higgins, Santiago, Chile; ^8^Institute of Medical Immunology, Charité–Universitätsmedizin Berlin, Corporate Member of Freie Universität Berlin, Humboldt Universität zu Berlin and Berlin Institute of Health, Berlin, Germany; ^9^Department of Clinical Research, Faculty of Infectious and Tropical Diseases, London School of Hygiene and Tropical Medicine, London, United Kingdom; ^10^Complex Chronic Diseases Program, British Columbia Women's Hospital and Health Centre, Vancouver, BC, Canada; ^11^CMAFcIO–Center of Mathematics, Fundamental Applications and Operations Research, Faculdade de Ciências, Universidade de Lisboa, Lisboa, Portugal

**Keywords:** disease trigger, cutoff value, stratification, Epstein-Barr virus, human cytomegalovirus, varicella-zoster virus, human herpesvirus-6, herpes simplex virus 1 and 2

## Abstract

The evidence of an association between Myalgic Encephalomyelitis/Chronic Fatigue Syndrome (ME/CFS) and chronic herpesviruses infections remains inconclusive. Two reasons for the lack of consistent evidence are the large heterogeneity of the patients' population with different disease triggers and the use of arbitrary cutoffs for defining seropositivity. In this work we re-analyzed previously published serological data related to 7 herpesvirus antigens. Patients with ME/CFS were subdivided into four subgroups related to the disease triggers: S_0_-42 patients who did not know their disease trigger; S_1_-43 patients who reported a non-infection trigger; S_2_-93 patients who reported an infection trigger, but that infection was not confirmed by a lab test; and S_3_-48 patients who reported an infection trigger and that infection was confirmed by a lab test. In accordance with a sensitivity analysis, the data were compared to those from 99 healthy controls allowing the seropositivity cutoffs to vary within a wide range of possible values. We found a negative association between S_1_ and seropositivity to Epstein-Barr virus (VCA and EBNA1 antigens) and Varicella-Zoster virus using specific seropositivity cutoff. However, this association was not significant when controlling for multiple testing. We also found that S_3_ had a lower seroprevalence to the human cytomegalovirus when compared to healthy controls for all cutoffs used for seropositivity and after adjusting for multiple testing using the Benjamini-Hochberg procedure. However, this association did not reach statistical significance when using Benjamini-Yekutieli procedure. In summary, herpesviruses serology could distinguish subgroups of ME/CFS patients according to their disease trigger, but this finding could be eventually affected by the problem of multiple testing.

## Introduction

Myalgic Encephalomyelitis/Chronic Fatigue Syndrome (ME/CFS) is a complex disease with unknown cause whose patients experience persistent fatigue that cannot be alleviated by rest and suffer from post-exertional malaise upon minimal physical and/or mental activity (1). Disease prevalence has been estimated around 0.4% after pooling data from different epidemiological studies (2). However, this estimate might be conservative (3, 4) due to poor societal recognition of the disease including amongst health professionals (5), the inexistence of an objective disease-specific biomarker for the corresponding diagnosis (6), a small number of well-designed epidemiological studies (7), and limited funding opportunities for more comprehensive and integrative research (8).

The etiology of ME/CFS and its pathogenesis remains a topic under intense debate with the proposal of many competing hypotheses (9–16). However, there is a general consensus that the disease could be initiated by a combination of genetic predisposing factors (17–20) and environmental triggers (e.g., exposure to toxins, chronic emotional and physical stress) (10, 21). In this regard, a large proportion of patients report an acute infection at the onset of their symptoms (22, 23). Herpesviruses such as the Epstein-Barr virus (EBV) and the human herpesvirus-6 (HHV6) were considered to be the main candidates for the causative agents of ME/CFS due to their high prevalence in adults and their reactivation observed in patients (11, 24, 25). To understand the role of these viruses on ME/CFS, many serological investigations were conducted with inconclusive or even contradicting findings (24). Possible reasons for this contrasting evidence could be related to disease misclassification and selection bias (26, 27), the necessity of dividing patients into different subtypes (28), the low number of patients recruited (6), or differences in the antigen and experimental assays used (29). An additional but often ignored reason is that serological studies are typically based on arbitrary cutoff values for identifying seropositive individuals or high antibody responders, as illustrated in two serological studies on herpesviruses (30, 31).

Recently, the analysis of serological data from the United Kingdom ME/CFS Biobank (UKMEB) did not find any association between ME/CFS and the presence of antibodies against chronic infections by different herpesviruses (32). In this work, we re-analyzed these data by dividing the ME/CFS patients into four subgroups related to non-infection vs. infection disease triggers. We also performed a sensitivity analysis of the association between ME/CFS and each herpesvirus as a function of the cutoff defining seropositivity.

## Materials and Methods

### Study Participants

All study participants are part of the UKMEB as described before (33). In summary, the data refer to a cohort of 226 patients with ME/CFS and 99 healthy controls (HC); note that the sample size of the healthy controls is in line with the ones used for this group by current serological studies on the role of herpesviruses on ME/CFS, as summarized elsewhere (24). At biobank enrollment, patients had to fill in a symptom's assessment questionnaire in which they were asked a specific question about whether they had an infection at the disease onset. This question had four categories of response, which we used to divide the patients into the following subgroups: subgroup S_0_– she/he did not know whether she/he had an infection at the disease onset (*n* = 42, 18.5%); subgroup S_1_– she/he did not have an infection at the disease onset (*n* = 43, 18.9%); subgroup S_2_– she/he had an infection at the disease onset, but this infection was not confirmed with a lab test (*n* = 93, 41.0%); subgroup S_3_– she/he had an infection at the disease onset and this infection was confirmed with a lab test (*n* = 48, 21.1%). In the participant questionnaire, patients were also asked to narrate the factors that could have triggered or contributed to the disease. Given that this was an open question, we only performed a brief description of the respective responses.

All individuals had age between 18 and 60 years old. Patients with ME/CFS were referred for a possible inclusion in the UKMEB by general practitioners working in the National Health System (NHS) of the United Kingdom. The respective diagnosis was confirmed using the 1994 Centers for Disease Control and Prevention (CDC) (34) or the 2003 Canadian Consensus Criteria (35) by the UKMEB dedicated clinical research team, according to their designed clinical protocol (36). Participants were excluded if they were taking any anti-viral drug or any medication that could alter their immune function in the three preceding months. Healthy controls were either family members or friends of the recruited patients with ME/CFS, or they were volunteers recruited by advertisement within Higher Education Institutions. Detailed information about exclusion and inclusion criteria of the UKMEB and additional information about recruitment and sample processing can be found elsewhere (36, 37).

### Herpesviruses Serology

Serological data and the respective laboratory procedures were previously described in the original study (32). However, given that the main focus of this early study was cellular immunology, the description of herpesviruses serology was kept to a minimum. We have now provided some additional details.

The following commercial ELISA assays from Demeditec Diagnostics (Kiel, Germany) were used to quantify the plasma concentrations of IgG antibodies against the following viruses: the human cytomegalovirus (CMV; Prod. Ref. DECMV01), EBV-VCA antigen (Prod. Ref. DEEBVG0150), EBV-EBNA1 antigen (Prod. Ref. DE4246), herpes simplex virus-1 (HSV1; Prod. Ref. DEHSV1G0500), herpes simplex virus-2 (HSV2; Prod. Ref. DEHSV2G0540), Varicella-Zoster virus (VZV; Prof. Ref. DEVZVG0490). The commercial ELISA-VIDITEST from VIDIA (Vestec, Czech Republic) was used for the IgG quantification against HHV6 (Prod. Ref. ODZ-235).

Antibody quantification was expressed in arbitrary units per milliliter (U/ml). According to manufacturer's instructions, seropositivity was considered for all samples with concentration ≥ 12 U/ml for HSV1, HSV 2, VZV, CMV and EBV antigens. Likewise, individuals with antibody concentrations against HHV6 ≥ 12.5 U/ml were considered seropositive.

### Statistical Analysis

To compare the age of the participants, the age of disease onset, and the disease duration of different study groups and/or subgroups of ME/CFS patients, we used the non-parametric Kruskal-Wallis test. To compare the gender distribution in the same subgroups, we applied the Pearson's *χ*^2^ test for testing independence in two-way contingency tables. For simplicity of the analysis, we only reported frequencies and the respective percentages of different disease triggers in the subgroups of ME/CFS that mentioned the occurrence of such triggers.

We previously performed thorough analyses of different cutoff values for seropositivity to each viral antigen (38, 39). These earlier analyses were based on the comparison and the selection of different scale mixtures of skew-normal distributions and four different criteria to define seropositivity. In accordance with a sensitivity analysis, instead of selecting a fixed cutoff, we here allowed this cutoff to vary between 10 U/ml and 100 U/ml with a lag of 1 U/ml. For each cutoff of a given antibody, we first estimated the unadjusted seropositivity odds ratio (OR) and the area under the Receiver Operating Characteristic curve (AUC) between each ME/CFS subgroup and the healthy controls using a logistic regression model in which seropositivity status of the individuals and a group indicator were the outcome and the covariate, respectively.

We then adjusted these ORs and AUCs using a similar logistic regression model but including age, gender, and a group indicator variable as the respective covariates. In both unadjusted and adjusted analyses, the effect of healthy controls was set as the reference of the group indicator variable. We calculated the *p*-values of the Wald's score test to assess the significance of different log-ORs of each subgroup of ME/CFS in relation to the group of healthy controls. The significance level of each executed test was set at 5%. In Supplementary File, one can find a detailed description of the likelihood function of the regression models used and how the ORs (or the log-ORs) are related to the parameters of these models.

To investigate the impact of multiple testing on the results, we adjusted the raw *p*-values of the Wald's score tests in order to ensure a false discovery rate of 5%. With this purpose, we used the Benjamini-Hochberg (BH) and Benjamini-Yekutieli (BY) procedures under the assumption of independent and positively correlated tests, respectively (40, 41). In this analysis, adjusted *p* < 0.05 were indicative of statistically significant associations.

Finally, we estimated the statistical power of the detected associations using a parametric Bootstrap approach (42). For each antibody, we used the following algorithm: (i) determine the optimal seropositivity cutoff by maximizing the likelihood ratio statistic as a function of this parameter when comparing the above logistic models with and without the group indicator covariate; (ii) generate the seropositivity data resulting from the optimal cutoff; (iii) estimate a logistic model including the group indicator as the only covariate (unadjusted analysis) or a logistic model including age, gender and group indicator variables as the respective covariates (adjusted analysis) using the seropositivity data obtained in (ii); simulate 1,000 data sets using the seropositive probability estimates obtained from models fitted in (iii); (iv) calculate the power of the association between seropositivity and each study group by the proportion of simulated data sets in which the association was deemed significant at the 5% significance level using the Wald's score test as described above.

The statistical analysis was conducted in the R software version 4.0.3. In particular, the estimation of the logistic regression models was done using the “glm” command and the analysis based on the AUC was conducted using the package “pROC” (43). In the multiple testing analysis, the raw *p*-values were adjusted by the BH and BY procedures using the package “MASS” (44). The corresponding scripts are available from the first or the corresponding author upon request.

## Results

### Basic Characterization of Study Participants

The four subgroups of ME/CFS had the same age distribution approximately (Kruskal-Wallis test, *p* = 0.30) with means of 44.6, 40.7, 43.3, and 40.9 years old for S_0_, S_1_, S_2_, and S_3_, respectively (Table 1). The respective mean ages of disease onset were 32.1, 30.2, 31.3, and 27.3 years old, while the mean disease durations were 12.7, 11.6, 12.1, and 13.5 years for the same subgroups. The differences in these variables were not statistically significant (*p* = 0.55 and 0.21, respectively). Similarly, the percentages of female patients ranged from 70.8% to 80.6%, but they were not statistically different (Pearson's *χ*^2^ test, *p* = 0.62).

**Table 1:** Basic characteristics of study participants where patients with ME/CFS were split into four subgroups according to the responses about their disease triggers in the symptoms' assessment questionnaire.

	**Healthy controls (*n* = 99)**	**Subgroups of ME/CFS patients**				**Comparison (*****P*****-values)**	
		**S_**0**_ (*n* = 42)**	**S_**1**_ (*n* = 43)**	**S_**2**_ (*n* = 93)**	**S_**3**_ (*n* = 48)**	**ME/CFS subgroups**	**ME/CFS subgroups + Healthy controls**
Female (%)	73 (73.7)	33 (78.6)	33 (76.7)	75 (80.6)	34 (70.8)	0.62	0.69
Mean age (IQR)	41.9 (32–51.5)	44.6 (35.0–53.8)	40.7 (28.0–52.0)	43.3 (35.0–53.0)	40.9 (32.0–50.3)	0.30	0.44
Mean age of disease onset (IQR)		32.1 (21.9–43.5)	30.2 (20.2–39.3)	31.3 (22.1–39.1)	27.3 (18.9–36.2)	0.21	-
Mean disease duration (IQR)	-	12.7 (5.30–17.90)	11.6 (4.2–15.9)	12.1 (5.5–16.5)	13.5 (6.0–19.2)	0.55	-
**Disease severity at recruitment**							
Mild/moderate (%)	-	38 (90.5)	39 (90.7)	64 (68.8)	34 (70.8)	0.003	-
Severely affected (%)	-	4 (9.5)	4 (9.3)	29 (31.2)	14 (29.2)	-	-
**Number of self-reported disease triggers**							
Single	-	-	19 (44)	52 (56)	32 (67)	<0.001^a^	-
Multiple	-	-	10 (23)	35 (38)	11 (23)	-	-
Missing	-	-	14 (33)	6 (6)	5 (10)	-	-

*S_0_, Do not know; S_1_, Non-infection trigger; S_2_, An infection trigger but not confirmed with a lab test; and S_3_, An infection trigger confirmed with a lab test*.

a *Pearson's *χ*^2^ test including the missing as a category for the number of disease factors/triggers*.

Overall, the percentage of severely affected patients significantly differed among the subgroups (Pearson's *χ*^2^ test, *p* = 0.003). In particular, the percentage of these patients in S_0_ and S_1_ was approximately 9%. This value was in clear contrast with the 30% of severely affected patients belonging to S_2_ and S_3_, both groups related to infection triggers.

In terms of the number of narrated disease factors or triggers reported in the participant's questionnaire, the subgroup S_1_ had the lowest percentage of patients reporting a single factor or trigger for their disease (44%) when compared to infection-related subgroups S_2_ and S_3_ (56 and 67%, respectively; Table 1). The same subgroup was the one with the highest percentage of missing data to this question (33% for S_1_ vs. 6 and 10% for S_2_ and S_3_, respectively). Overall, the distribution of the number of reported disease factors or triggers was significantly different among subgroups S_1_, S_2_, and S_3_ (Pearson's *χ*^2^ test, *p* < 0.001) mostly due to differences in the amount of missing data.

These subgroups of ME/CFS patients were well matched for gender and age with respect to the healthy control group (Pearson's *χ*^2^ and Kruskal-Wallis tests, *p* = 0.69 and 0.44, respectively).

### Disease Factors or Triggers Narrated by Different Subgroups of ME/CFS Patients

When the 184 patients belonging to the subgroups S_1_, S_2_, and S_3_ were asked to narrate the factors or triggers of their disease in the participant questionnaire, 103 (56%) and 56 (30%) of them reported single and multiple factors (or triggers), respectively. However, 25 patients (14%) did not mention any specific trigger or factor contributing to their disease. In total, there were 14 distinct categories of disease triggers narrated by the patients. These categories were consistent to the ones reported by previous epidemiological studies about possible triggers of ME/CFS (22, 23).

The following non-infection factors or triggers were mentioned by patients mostly belonging to the subgroup S_1_: stress subdivided into general anxiety (9%, *n* = 20), personal (8%, *n* = 18) or professional-related stress (5%, *n* = 11); accidents/injuries/surgeries (5%, *n* = 11); pregnancy, childbirth and other problems related to women's reproduction system (3%, *n* = 6), and other non-infection triggers (Table 2). The remaining factors were related to microbial infections and/or infectious diseases: upper respiratory tract infections – glandular fever (GF), tonsillitis, EBV infections, or throat infection (21%, *n* = 48); lower respiratory tract infections – chest infection or pneumonia (4%, *n* = 10); flu- or cold-like illnesses (11%, *n* = 26); gastrointestinal problems and related infections (4%; *n* = 9); and tropical infectious diseases – Dengue fever and schistosomiasis (1%, *n* = 3); and other viral or bacterial infection, and unspecified infections (22%, *n* = 51). Note that 6 patients from subgroup S_1_ mentioned an infection in the narrative question about the factors or triggers of their disease. However, the same patients also reported other possible non-infection triggers, such as trauma, bereavement, and stress. We speculate that these patients attributed a higher likelihood to these non-infection disease triggers when answering the related question in the symptoms' assessment questionnaire. Interestingly, patients belonging to the subgroup S_3_ reported the highest percentage of disease factors or triggers consistent with an EBV infection (46%, *n* = 22). Patients from subgroup S_2_ also self-reported a high frequency of EBV-related factors or triggers (27%, *n* = 25), but closely matched by a flu-like infection or illness (22%, *n* = 20).

**Table 2:** Frequency and the respective percentage within brackets of specific disease factors or triggers narrated by patients from the subgroups S_1_ (*n* = 43), S_2_ (*n* = 93), and S_3_, (*n* = 48) in the participant's questionnaire.

**Narrated disease trigger**	**Subgroups of ME/CFS patients**			**Total (%)**
	**S_**1**_ (%)**	**S_**2**_ (%)**	**S_**3**_ (%)**	
Glandular Fever; tonsilitis; EBV infection	1 (2)	25 (27)	22 (46)	48 (21)
Respiratory infection; pneumonia	1 (2)	6 (6)	3 (6)	10 (4)
Flu-like infection or illness	2 (5)	20 (22)	4 (8)	26 (11)
Gastrointestinal infection	0 (0)	6 (6)	3 (6)	9 (4)
Tropical infections	0 (0)	1 (1)	2 (4)	3 (1)
Other infections including unspecified viral infections	2 (5)	33 (35)	13 (27)	51 (22)
General stress or anxiety	6 (14)	11 (12)	3 (6)	20 (9)
Stress due to personal events	9 (21)	6 (6)	3 (6)	18 (8)
Stress at work or school	4 (9)	5 (5)	2 (4)	11 (5)
Vaccinations	0 (0)	4 (4)	6 (12)	10 (4)
Chemical exposure	1 (2)	6 (6)	0 (0)	7 (3)
Accidents/Injuries/Surgeries	7 (16)	2 (2)	2 (4)	11 (5)
Pregnancy/Childbirth/Postnatal/ Hysterectomy/Endometriosis	6 (14)	0 (0)	0 (0)	6 (3)
Other	4 (9)	6 (6)	0 (0)	7 (3)

### Serological Data Analysis by Subgroup of ME/CFS Patients

**Figure 1:**
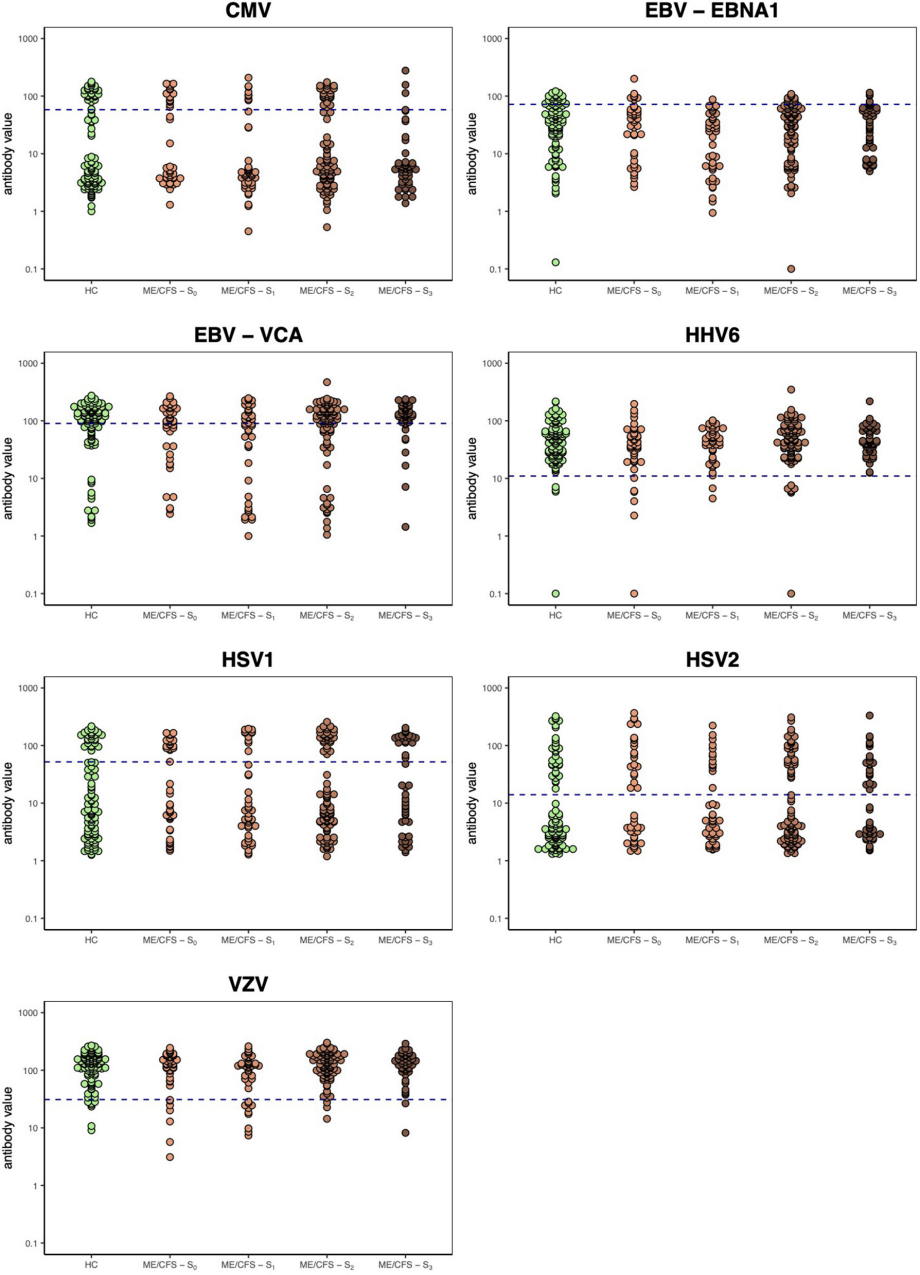
Herpesvirus serology data per study group including the four ME/CFS subgroups. Horizontal dashed lines represent the optimal seropositivity cutoff for the unadjusted analysis according to the maximization of likelihood ratio statistic for testing the significance of the group indicator covariate in the logistic models (see Supplementary Figure 1).

We then compared the serological data of these ME/CFS subgroups of patients with the group of healthy controls (Figure 1). In this analysis, we intended to investigate the impact of cutoff on the resulting seropositivity OR and AUC between each subgroup of ME/CFS patients and the group of healthy controls.

**Figure 2:**
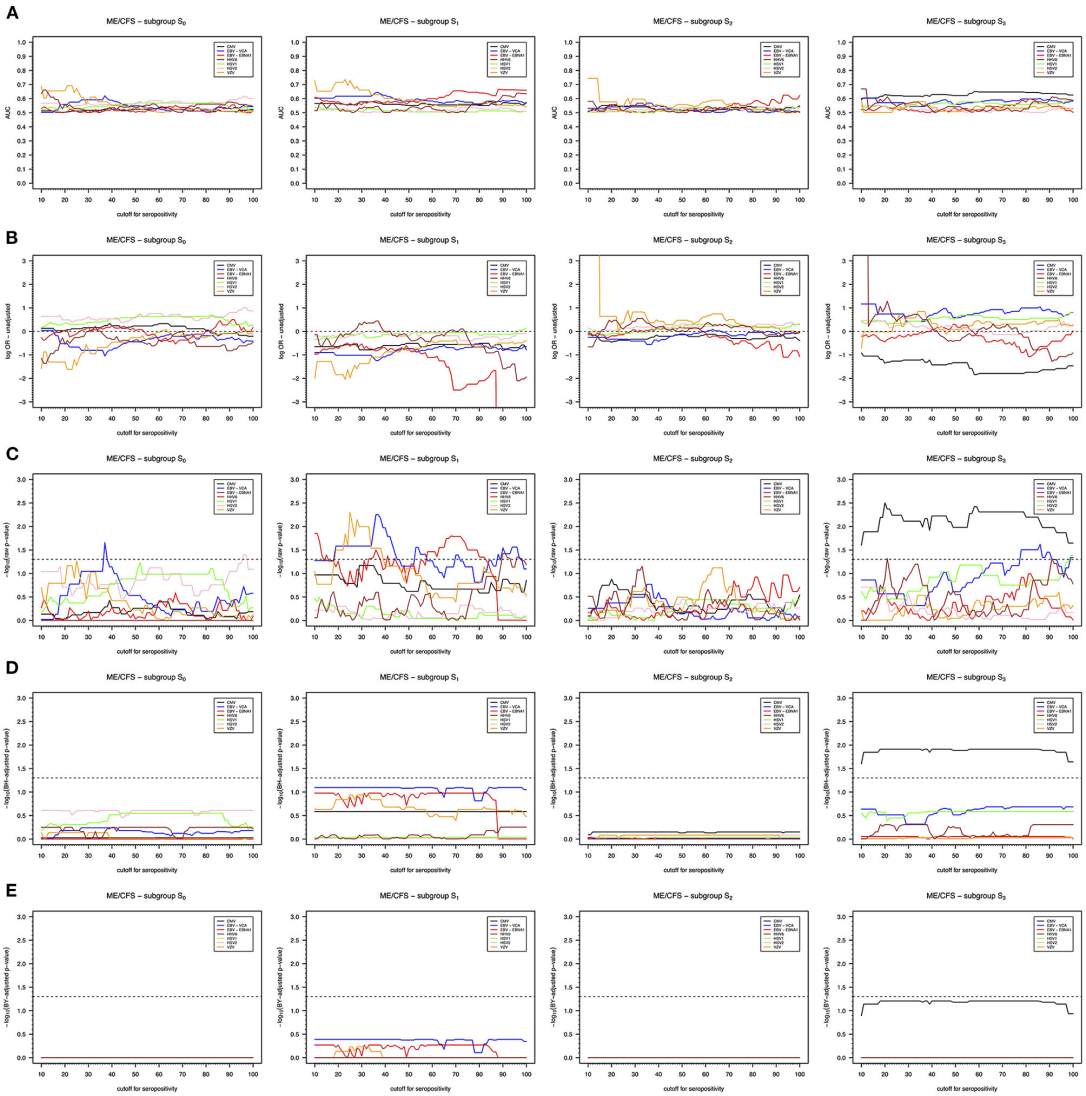
Unadjusted association analysis of seropositivity to different herpesvirus antigens as function of the cutoff defining seropositivity. **(A)** AUC of the probabilities of being seropositive estimated in each subgroup of ME/CFS patients when compared to the same probabilities estimated in the group of healthy controls. **(B)** Log-ORs of being seropositive in each subgroup of ME/CFS patients in relation to the group of healthy controls. **(C)** Raw *p*-values of Wald's score test for the significance of log-ORs. **(D)** The corresponding BH-adjusted *p*-values. **(E)** The corresponding BY-adjusted *p*-values. For convenience, raw and adjusted *p*-values were transformed as –log_10_(*p*-value). The dashed lines in the respective plots represent the threshold referring to the 5% significance level [i.e., –log_10_(0.05); **C**] or the 5% false discovery rate. **(D,E)** Cutoff values in which –log_10_(*p*-values) are above these thresholds provided evidence for significant associations.

With respect to unadjusted analysis, the AUCs were in most cases estimated between 0.50 and 0.60 (Figure 2A). This finding suggested that serological data had limited predictive power to discriminate the seropositivity of subgroups of ME/CFS patients from that of healthy controls. The highest estimated AUC was approximately 0.75 for VZV when comparing the seropositivity of the subgroup S_2_ to healthy controls using cutoffs below 15 U/ml.

According to the OR estimates, we could not find any significant association of subgroups S_0_ and S_2_ with herpesviruses serology (Figures 2B,C). The only exception was a putative association between the subgroup S_0_ and the antibodies against EBV-VCA using a cutoff of 37 U/ml. Interestingly, before correcting for multiple testing, we found significant negative associations (i.e., negative log-ORs) between the subgroup S_1_ and antibodies against EBV-VCA, EBV-EBNA1, and VZV depending on the cutoff used (Figures 2B,C). These negative associations suggested decreased seroprevalences to these herpesviruses in this subgroup when compared to the group of healthy controls. When controlling for multiple testing, these associations were not considered statistically significant using either BH or BY procedures (Figures 2D,E).

We also found a significant negative association between subgroup S_3_ and CMV seropositivity (Figures 2B,C), which suggested decreased antibody levels in this subgroup of patients in relation to group of healthy controls. The corresponding AUC was estimated around 0.65 for most of cutoffs (Figure 2A). This association was consistent across the range of cutoffs specified for the analysis and even after controlling for multiple testing using the BH procedure based on the assumption of independent tests (Figure 2D). However, the statistical significance of the association was lost after using the BY procedure based on the assumption of positively correlated tests (Figure 2E).

Similar findings were obtained when adjusting for possible confounding effects of age and gender (Supplementary Figures 2A–E). This agreement between unadjusted and adjusted analyses can be explained by a good matching between the different ME/CFS subgroups of patients and healthy controls in terms of age and gender (Table 1). However, the significance of adjusted ORs was slightly reduced due to these putative confounding factors (Supplementary Figure 2C).

Finally, we estimated the statistical power related to the identified associations using the optimal seropositivity cutoff for each herpesvirus antibody. For the unadjusted analysis, these optimal cutoffs varied from 11 U/ml to 90 U/ml (Supplementary Figure 1 and Supplementary Table 1). Similar optimal cutoffs were obtained for the analysis adjusting for age and gender (Supplementary Figure 3 and Supplementary Table 1) with the exception of EBV-EBNA1 for which the optimal cutoffs were 72 U/ml and 88 U/ml for the unadjusted and adjusted analyses, respectively. The maximum power (~90%) was obtained for the association between CMV seropositivity and ME/CFS subgroup S_3_ in either unadjusted or adjusted analyses (Supplementary Figure 4). A high power (~75%) was also obtained for the associations between VZV seropositivity and ME/CFS subgroup S_1_. The remaining associations between each study group and herpesvirus seropositivity had a power that did not exceed 60%. In conclusion, the manufacturer's seropositivity cutoffs were not the most adequate to maximize the chance of finding an association of ME/CFS subgroups with the herpesviruses serology and only three associations between the study groups and herpesviruses seropositivity had a high statistical power.

## Discussion

In contrast with the original study where we could not find differences related to herpesviruses serology between healthy controls and ME/CFS patients divided according to their disease severity (32), our re-analysis of the same data identified two subgroups of ME/CFS patients (S_1_ and S_3_) in which such differences are now statistically significant. This new finding was only possible due to the stratification of patients according to a question related to the occurrence of an infection at disease onset together with a sensitivity analysis of the seropositivity cutoff used. Patients' stratification or subtyping was performed in line with past recommendations for ME/CFS research (28). Following this recommendation, we previously performed an immunological investigation based on a stratification of ME/CFS patients according to the severity of their symptoms (32). By using this stratification, we showed perturbations in the T-cell compartment, namely, in effector CD8^+^ T cells and in the mucosal-associated invariant T cells. In another study using similar stratification of the samples from the UKMEB, the levels of the cellular stress biomarker GDF15 were found to be increased in severely affected patients at different time points (45). We speculate that other immunological perturbations could be detected if our alternative stratification could have been used. This investigation will be carried out in the near future.

In line with our findings, evidence has been emerging that the occurrence of an acute infection at the onset of disease symptoms is indeed a key stratifying factor to detect genetic and immunological differences between subgroups of ME/CFS patients when compared to healthy controls (17, 46). However, the simplistic approach of dividing patients according to non-infection and infection triggers might not be sufficient to obtain relatively homogeneous subgroups of ME/CFS patients, which affects the statistical power to detect any disease-specific effects. Besides the limited choice of antibodies against different herpesvirus-related antigens, the large heterogeneity in infectious triggers seems a possible explanation for the lack of association between the subgroup S_2_ and herpesviruses seropositivity. Notwithstanding not having their infection trigger confirmed in the lab, patients from this subgroup reported the highest proportion of flu-like illnesses, which could have been caused by the influenza virus, the rhinovirus, or the respiratory syncytial virus (47). It is then conceivable that these patients exhibit different pathological mechanisms of ME/CFS according to the causative virus, some of which without any direct impact on the antibody responses against herpesviruses. To overcome these problems, we recommend the collection of infection-trigger data as detailed and accurate as possible.

Our most consistent association was obtained between CMV seropositivity and patients from the subgroup S_3_. These patients tended to be less seropositive to this herpesvirus when compared to healthy controls, irrespective of the seropositivity cutoff value used. Previously, different serological investigations did not provide conclusive evidence for the role of CMV on ME/CFS pathogenesis, as reviewed elsewhere (24). The lack of or the use of an inadequate stratification could also explain these past findings. In this regard, the unveiled association was obtained in a subgroup in which the accuracy of the reporting might be the highest, because the disease-triggering infections were supposedly confirmed in the lab. However, we cannot ignore the fact that this subgroup has a large fraction of patients whose disease trigger was related to an EBV infection, one of the most reported causative agents of ME/CFS. Therefore, it is possible that our finding resulted from a coincidence between a low-resolution patient's stratification and a random enrichment of a specific infection trigger in one of the subgroups.

A supposedly decreased seropositivity (or antibody levels) to CMV in an EBV-infection trigger could be explained by the hyperregulation hypothesis (11). According to this hypothesis, a possible pathological mechanism of ME/CFS is related to an expansion of regulatory CD4^+^ T cells (Tregs) driven by an autoimmune response against a viral antigen that mimics a self-antigen. This expansion of Tregs upon herpesvirus infection or reactivation locks the (adaptive) immune system in an active state of hyperregulation where different infections are more difficult to be cleared from the body. Frequent infections are in fact reported by patients with ME/CFS (33). The question is then how the expansion of Tregs could affect antibody responses against CMV. The so-called follicular Tregs might hold the answer to this question. These specialized Tregs are derived from Treg precursors with the ability to migrate to germinal center reactions (GCRs) to inhibit the respective antibody production and antibody maturation (48). In particular, experiments with animal models demonstrated that the amount of IgG antibodies against different foreign antigens is increased in immunized mice depleted of follicular Tregs (49, 50). In this line of thought, it is reasonable to assume that an increased proportion of Tregs in ME/CFS could be translated into an increased proportion of follicular Tregs. This increase could in turn decrease the antibody production derived from GCRs. We can then hypothesize that an EBV infection triggered an autoimmune response that disrupted the normal balance between Tregs and effector CD4^+^ T cells; a peptide of the viral EBNA6 was found to share a high sequence homology with the human lactoperoxidase and thyroid peroxidase (30). The disruption of this balance could lead to an increase of both natural and follicular Tregs. A possible consequence of this increase is a diminished antibody production against a posterior CMV infection or reactivation. Note that several peptides from CMV were also found as putative candidate for molecular mimicry with human proteins (51). Similar to the situation of immunosuppression, a reduction in the humoral immunity against CMV would render ME/CFS patients more susceptible to a possible reactivation of this virus (52). It is worth noting that the role of follicular Tregs was never investigated in ME/CFS patients.

Another interesting finding is the possible association between the subgroup S_1_ and EBV and VZV seropositivity. This subgroup refers to patients who reported non-infectious triggers, mostly related to stressful or stress-related events. It is also a group where ME/CFS was triggered in many women who had problems during and after pregnancy, had difficult childbirth or had disorders related to women's reproduction system. In line with this finding, stressful conditions and events such as the ones experienced by astronauts increase the chance of herpesvirus reactivation, specifically, EBV, VZV and CMV (53). Reactivation of latent herpesvirus infections could be explained by an increase in production of stress-related hormones together with an inflammatory cytokine signature that debilitates the immune system. This subgroup is then expected to have a higher prevalence of active herpesvirus infections than the remaining subgroups of ME/CFS patients and healthy controls. Given that this subgroup could represent <50% of the patients (22, 23), it is likely to have insufficient statistical power to detect any differences in herpesvirus reactivation rates between ME/CFS and healthy controls even in the case of a proper stratification of the patients' populations. This limitation is yet another reason that could explain the inconsistent findings on herpesvirus reactivation across many studies on ME/CFS.

We did not find any association between the subgroup S_0_ and herpesviruses seropositivity. This subgroup represented 18.5% of the patients' cohort, a value compatible with the percentages of patients that did not report any disease triggering event from past epidemiological studies [10%, (22); 24%, (54)]. The sample size of this subgroup was not very large and, therefore, we cannot rule out that our lack of associations could be simply due to insufficient statistical power to detect putative associations between this subgroup and herpesviruses seropositivity.

In our association analysis, we allowed the seropositivity cutoff to vary within a given range of possible values, similarly done in a recent study of molecular mimicry between Anoctamin 2 and EBNA1 in multiple sclerosis (55). This analytical approach seems reasonable given the difficulty to choose the best seropositivity cutoff among the different criteria and methods available, as illustrated in the earlier analyses of the same data (38, 39). This approach is also in line with several discussions about seropositivity estimation and the sensibility to use a fixed cutoff (56–59). However, varying the cutoffs defining seropositivity might increase the chance of false positives due to the multiple testing problem. We attempted to overcome this problem by controlling the false discovery rate, but the small sample size of each subgroup of ME/CFS did not allow to reach statistical significance of the detected associations when using the BY procedure based on the assumption of multiple positively correlated tests. On the other hand, our power calculations suggested a high probability of detecting some of the associations under the assumption that they were actually true. Therefore, the correct application of cutoff-varying approach should include a thorough assessment of a putative multiple testing problem together with a power calculation in order to assess the statistical consistency of the findings.

As final remarks, we should also note that cutoffs for detecting associations between herpesviruses and ME/CFS might vary from one lab to another and with the serological kits used. In addition, a high cutoff for the data might not define seropositivity *per se*, but rather a high antibody response whose detection could be the primary objective of the analysis (30, 31). The use of a high cutoff is also in accordance with a modeling approach where seropositivity might be subdivided into different levels (60–62). Therefore, our sensitivity-like approach seems to have the capacity to detect further serological associations beyond the ones based on the classical seroprevalence. Such a capacity could increase the chance of reaching scientific reproducibility. We then recommend the routine use of this approach in future serological investigations of ME/CFS.

## Data Availability Statement

The raw data supporting the conclusions of this paper will be made available from Eliana M. Lacerda upon request.

## Ethics Statement

The studies involving human participants were reviewed and approved by London School of Hygiene & Tropical Medicine (LSHTM) Ethics Committee (Ref. 6123) and the National Research Ethics Service (NRES) London-Bloomsbury Research Ethics Committee (REC ref. 11/10/1760, IRAS ID: 77765). The patients/participants provided their written informed consent to participate in this study.

## Author Contributions

NS conceived this research. HM and NS supervised this research. J-SL and JC generated the serology data. AG and EL compiled and curated the data related to ME/CFS triggers. EL and LN were responsible for data collection of the UKMEB. TD, AG, JA-A, and NS performed the data analysis. FW, CS, LN, and HM helped in data interpretation. TD, AG, and NS wrote the manuscript. All authors have revised, read, and approved the final version of the manuscript.

## Conflict of Interest

The authors declare that the research was conducted in the absence of any commercial or financial relationships that could be construed as a potential conflict of interest.
