# Supplementary material for: Herpesviruses Serology Distinguishes Different Subgroups of Patients From the United Kingdom Myalgic Encephalomyelitis/Chronic Fatigue Syndrome Biobank
Source: Front Med (Lausanne). 2021 Jul 5;8:686736. doi: 10.3389/fmed.2021.686736 (PMC8287507; doi:10.3389/fmed.2021.686736)
Supplement: Supplementary file 1 [file Data_Sheet_1.pdf]

Supplementary Material

1 DETAILED STATISTICAL DESCRIPTION OF THE ANALYSIS

Let us focus on the analysis of the antibody $j = 1, \ldots, 7$ and a generic cutoff for seropositivity $c = 10, \ldots, 100$. Let Y_{ij} be the random variable representing the respective antibody value of the i-th individual where $i = 1, \ldots, n$. Let X_{ijc} be the Bernoulli variable representing the corresponding serological status of i-th individual given the cutoff c. The respective probability mass function is given by

$$P\left[X_{ijc} = x | \pi_{ijc}\right] = \pi_{ijc}^{x} \left(1 - \pi_{ijc}\right)^{1-x}, x = 0, 1 \qquad \text{(S1)}$$

where $\pi_{ijc} = P[Y_{ij} > c]$. Under the assumption of independent distributions for X_{ijc}, the respective sampling distribution of $\mathbf{X}_{jc} = (X_{ijc}, i = 1, \ldots, n)$ can be written as

$$P\left[\mathbf{X}_{jc} = \mathbf{x}_{jc} | \mathbf{\Pi}_{jc}\right] = \prod_{i=1}^{n} \pi_{ijc}^{x_{ijc}} \left(1 - \pi_{ijc}\right)^{1-x_{ijc}}, \qquad \text{(S2)}$$

where $\mathbf{\Pi}_{jc} = \{\pi_{ijc}, i = 1, \ldots, n\}$ and $\mathbf{x}_{jc} = \{x_{ijc}, i = 1, \ldots, n\}$. This sampling distribution is mathematically equivalent to the likelihood function $\mathcal{L}(\mathbf{\Pi}_{jc} | \mathbf{x}_{jc})$ when the unknown parameters $\mathbf{\Pi}_{jc}$ are taken as (unknown) variables and the observed $\mathbf{x}_{jc}$ as constants.

For the unadjusted analysis in which we only included the effect of the subgroups, we used the following logistic regression model

$$\log \frac{\pi_{ijc}}{1 - \pi_{ijc}} = \alpha_{jc} + \beta_{0jc} S_i^0 + \beta_{1jc} S_i^1 + \beta_{2jc} S_i^2 + \beta_{3jc} S_i^3, \qquad \text{(S3)}$$

where S_i^0, S_i^1, S_i^2, and S_i^3 are dummy variables indicating whether the i-th individual belongs to subgroups S_0, S_1, S_2, and S_3, respectively; α_{jc} and $\beta_{kjc}, k = 0, 1, 2, 3$ are unknown parameters that were estimated via the 'glm' function available in the R software. Note that, if the i-th individual belongs to the group of healthy controls, then $S_i^0 = S_i^1 = S_i^2 = S_i^3 = 0$. In this formulation, it is easy to demonstrate that the odds ratio (OR) Δ_{kjc} of the probability an individual belonging to the subgroup S_k being seropositive and the same probability for a healthy control is given by

$$\Delta_{kjc} = e^{\beta_{kjc}}, k = 0, 1, 2, 3. \qquad \text{(S4)}$$

Equivalently,

$$\log \Delta_{kjc} = \beta_{kjc}, k = 0, 1, 2, 3. \qquad \text{(S5)}$$

Figure 2 shows the estimated log-ORs as function of antibody and cutoff under analysis.

For the adjusted analysis in which we included the effect of the subgroups plus the effect of age and gender, we extended the above model as follows

$$\log \frac{\pi_{ijc}}{1 - \pi_{ijc}} = \alpha_{jc} + \beta_{0jc} S_i^0 + \beta_{1jc} S_i^1 + \beta_{2jc} S_i^2 + \beta_{3jc} S_i^3 + \gamma_{jc} z_i + \gamma_{jc}^{*} z_i^{*}, \qquad \text{(S6)}$$

where z_i represents the age of the i-th individual and z_i^* is a dummy variable indicating if the same individual is a woman. The calculation of the log-odds ratio presented in Figure S2 follows as described above for the unadjusted analysis.

2 LIST OF ABBREVIATIONS

In the following supplementary figures and tables, we used the abbreviations as written in the main text:

- CMV — Cytomegalovirus;
- EBV — Epstein-Barr virus;
- HHV6 — Human herpesvirus-6;
- HSV1 — Herpes simplex virus 1;
- HSV2 — Herpes simplex virus 2;
- VZV — Varicella-Zoster virus .

3 FIGURES

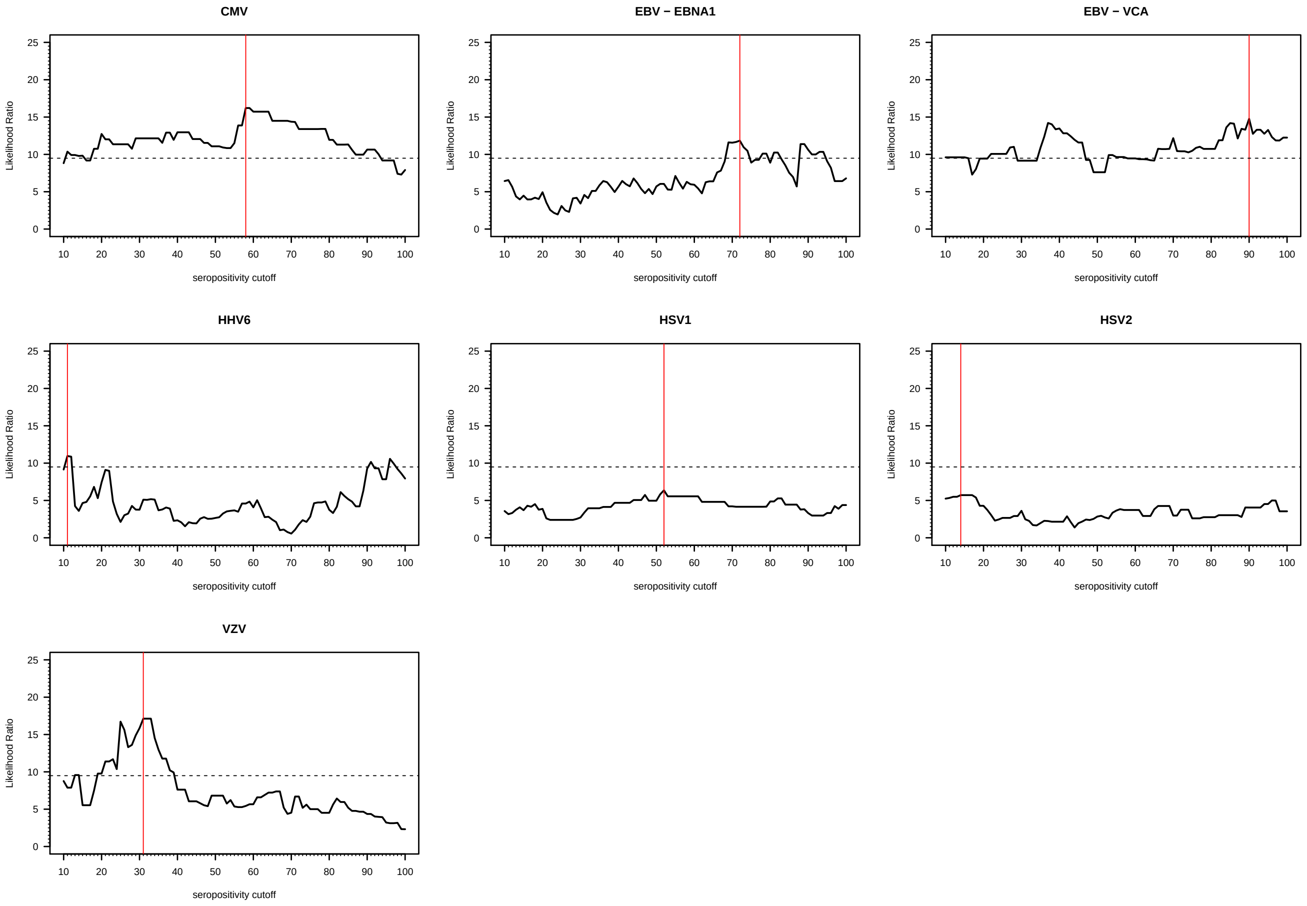

Figure S1. Seropositivity cutoff versus the likelihood ratio statistic for testing the significance of a group indicator covariate in the logistic models used in unadjusted analysis. The vertical red lines represent the optimal cutoff in which the maximization of the likelihood ratio statistic is achieved. The dashed horizontal lines represent the critical point of the likelihood ratio test. This critical point is defined by the 95% quantile of the χ^2 distribution with 5 degrees of freedom for the likelihood ratio statistic under the null hypothesis.

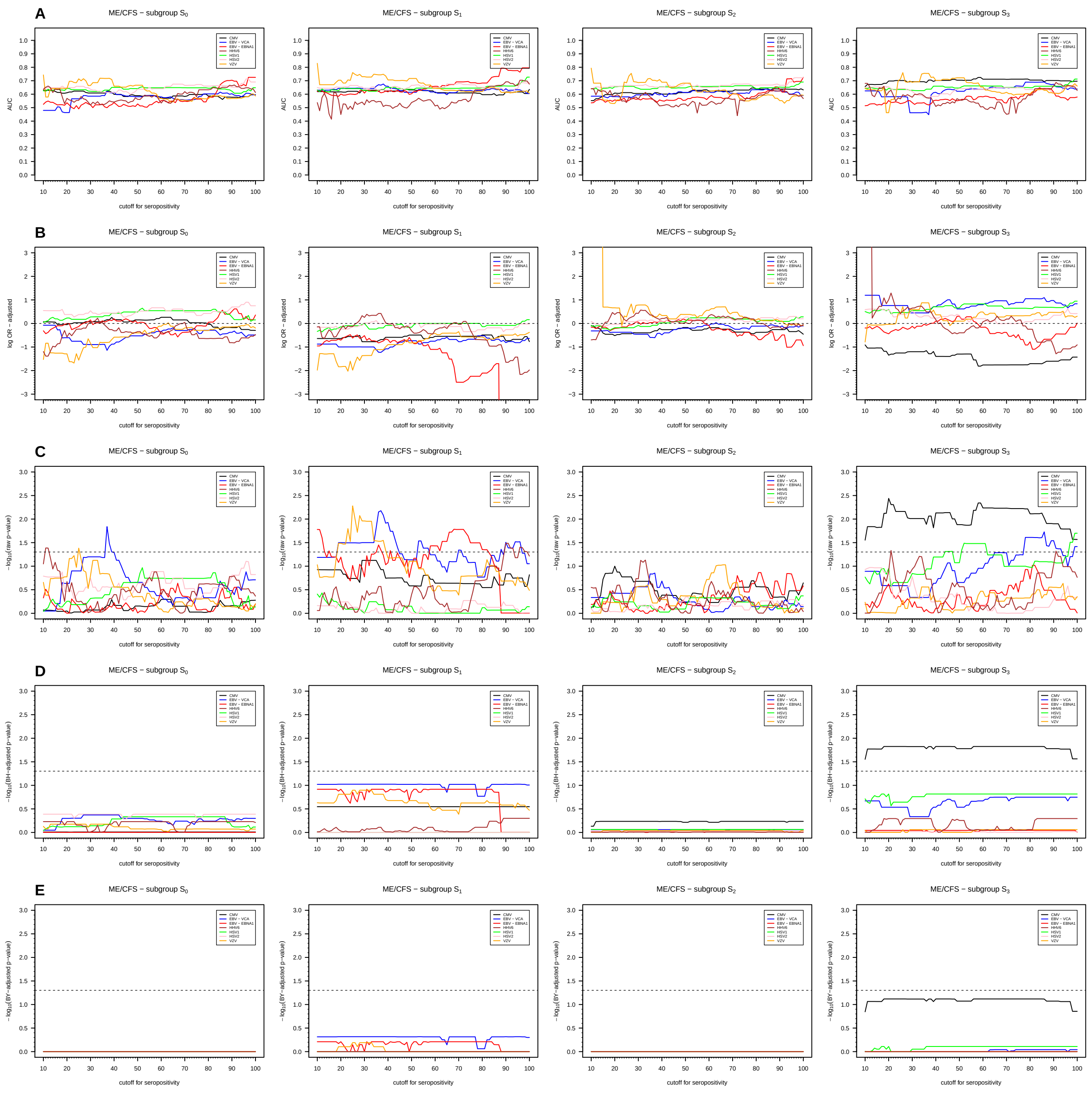

Figure S2. Age-gender adjusted association analysis of seropositivity to different herpesvirus antigens as function of the cutoff defining seropositivity. **A.** Area under the receiver operating characteristic curve (AUC) of the probabilities of being seropositive estimated in each subgroup of ME/CFS patients when compared to the same probabilities for the group of healthy controls; **B.** Logarithm of the odds ratios (log-ORs) of being seropositive in each subgroup of ME/CFS patients in relation to the group of healthy controls; **C.** Raw p-values of Wald's score test for the significance of log-ORs; **D.** The corresponding adjusted p-values using the Benjamini-Hochberg (BH) procedure; **E.** The corresponding adjusted p-values using the Benjamini-Yekutieli (BY) procedure. For convenience, raw and adjusted p-values were transformed as $-\log_{10}(\text{p-value})$. The dashed lines in the respective plots represent the threshold referring to the 5% significance level (i.e., $-\log_{10}(0.05)$; C) or the 5% false discovery rate (D and E). Cutoff values in which $-\log_{10}(\text{p-values})$ are above these thresholds provided evidence for significant associations..

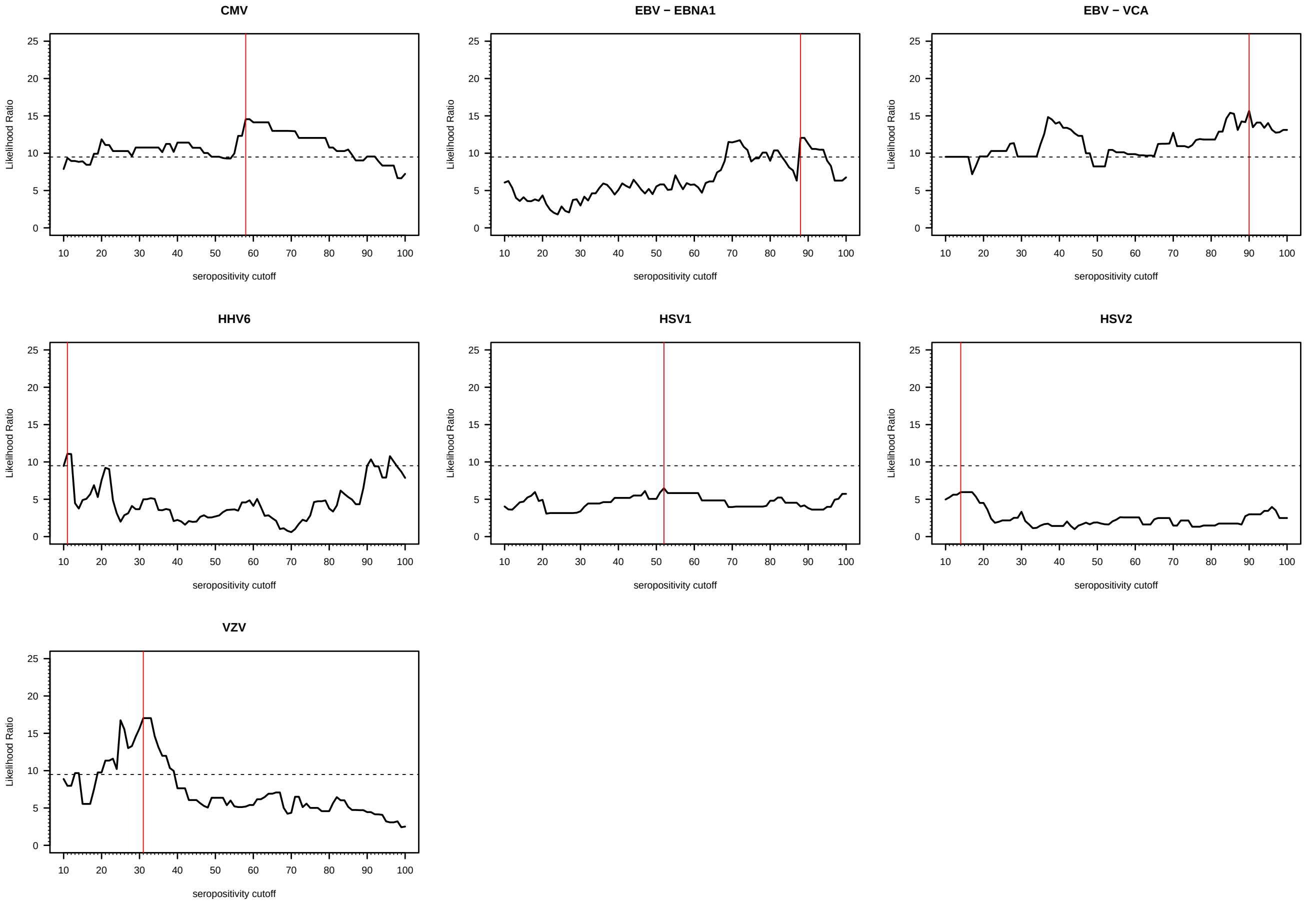

Figure S3. Seropositivity cutoff versus the likelihood ratio statistic for testing the significance of a group indicator covariate in the logistic models used in the analysis controlling for age and gender. See Figure S1 for further information.

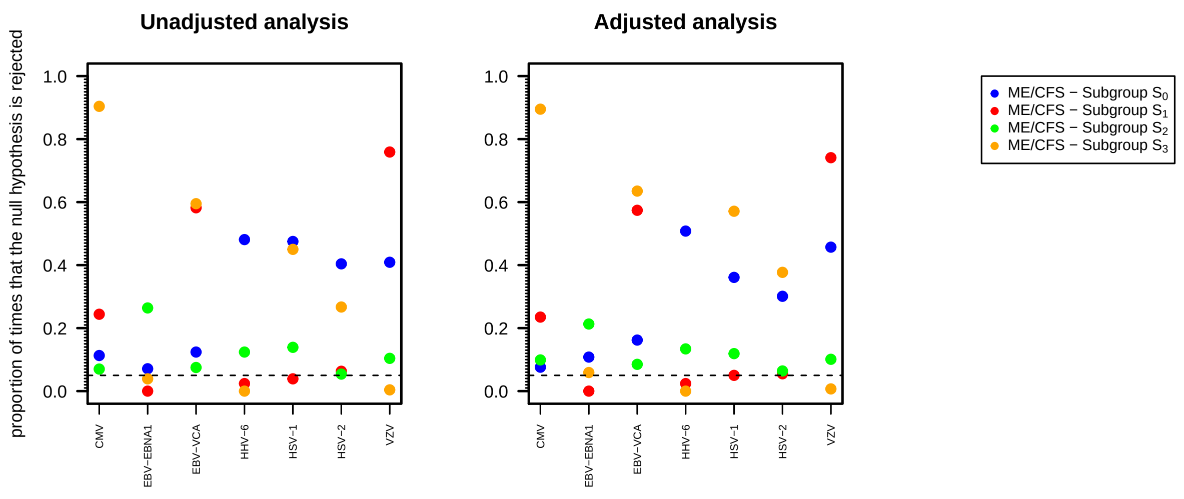

Figure S4. Statistical power to detect an association between each study group and the seropositivity to a given herpesvirus. Statistical power was estimated by the proportion of times when the log odds ratio between a given study group and healthy controls was deemed statistically significant at 5% significance level in 1000 simulated data sets from logistic models using the optimal cutoffs shown in Figures S1 and S3. Horizontal dashed lines represent the 5% significance level.

4 TABLES

Herpesvirus serology	Unadjusted analysis	Adjusted analysis
CMV	58	58
EBV-EBNA1	72	88
EBV-VCA	90	90
HHV6	11	11
HSV1	52	52
HSV2	14	14
VZV	31	31

Table S1. Optimal seropositivity cutoff for each herpesvirus antibody as shown in Figures S1 (unadjusted analysis) and S3 (age and gender adjusted analysis).
